# The strain-8 study: a multimodal scan–rescan assessment of myocardial strain repeatability

**DOI:** 10.1093/ehjimp/qyaf144

**Published:** 2025-11-14

**Authors:** Andrew Bell, Joseph Okafor, Momina Yazdani, Russell Franks, Jane Draper, Brian Campbell, Stamatis Kapetanakis, Amedeo Chiribiri, Alistair Young, Muhummad Sohaib Nazir

**Affiliations:** School of Biomedical Engineering and Imaging Sciences, King’s College London, 5th Floor, Beckett House, 1 Lambeth Palace Road, London SE1 7EU, UK; National Heart and Lung Institute, Imperial College London, London, UK; Department of Echocardiography, Royal Brompton and Harefield Hospitals, Guy’s and St Thomas’ Foundation Trust, London, UK; National Heart and Lung Institute, Imperial College London, London, UK; School of Biomedical Engineering and Imaging Sciences, King’s College London, 5th Floor, Beckett House, 1 Lambeth Palace Road, London SE1 7EU, UK; Department of Cardiology, Guys and St Thomas Hospital, London, UK; Department of Cardiology, Guys and St Thomas Hospital, London, UK; Department of Cardiology, Guys and St Thomas Hospital, London, UK; School of Biomedical Engineering and Imaging Sciences, King’s College London, 5th Floor, Beckett House, 1 Lambeth Palace Road, London SE1 7EU, UK; Department of Cardiology, Guys and St Thomas Hospital, London, UK; School of Biomedical Engineering and Imaging Sciences, King’s College London, 5th Floor, Beckett House, 1 Lambeth Palace Road, London SE1 7EU, UK; School of Biomedical Engineering and Imaging Sciences, King’s College London, 5th Floor, Beckett House, 1 Lambeth Palace Road, London SE1 7EU, UK; Department of Cardiology, Guys and St Thomas Hospital, London, UK; Department of Cardio-Oncology Service, Royal Brompton and Harefield Hospitals, Guy’s and St Thomas’ Foundation Trust, London, UK

## Abstract

**Aims:**

Myocardial strain is a powerful, non-invasive diagnostic and prognostic marker in patients with heart disease. However, its applicability is hindered by uncertain repeatability, particularly for segmental values. This study measures the repeatability of myocardial strain across eight imaging methods.

**Methods and results:**

In this prospective study, 20 healthy volunteers (aged 34 ± 8, 14 men) were recruited and scanned twice with eight strain imaging protocols: cardiac magnetic resonance (CMR) at 1.5T and 3T with cine, tagging, and displacement encoding with stimulated echoes (DENSE) sequences, and 2D and 3D echocardiography (Echo). Global and segmental strains were quantified from each scan. Inter-scan repeatability was assessed with the coefficient of variation (CoV), intraclass correlation coefficient, and Bland–Altman analysis.

**Results:**

Inter-scan repeatability of global strains ranged from excellent to fair (CoV ≤ 20%) depending on protocol. Using CMR feature tracking at 1.5T, relative global longitudinal strain (GLS) changes exceeding 11.2% are unlikely to be caused by measurement variability alone; this figure is 5.5% for 2D echocardiography. Segmental strain values frequently had poor repeatability (CoV > 20%), particularly for longitudinal and radial strains.

**Conclusion:**

Imaging protocols including CMR and Echo can measure global strain parameters with fair repeatability, but segmental strain values are unreliable. Future work should aim to improve the repeatability of segmental strain values, particularly longitudinal strain.

## Graphical Abstract

**Graphical Abstract:**
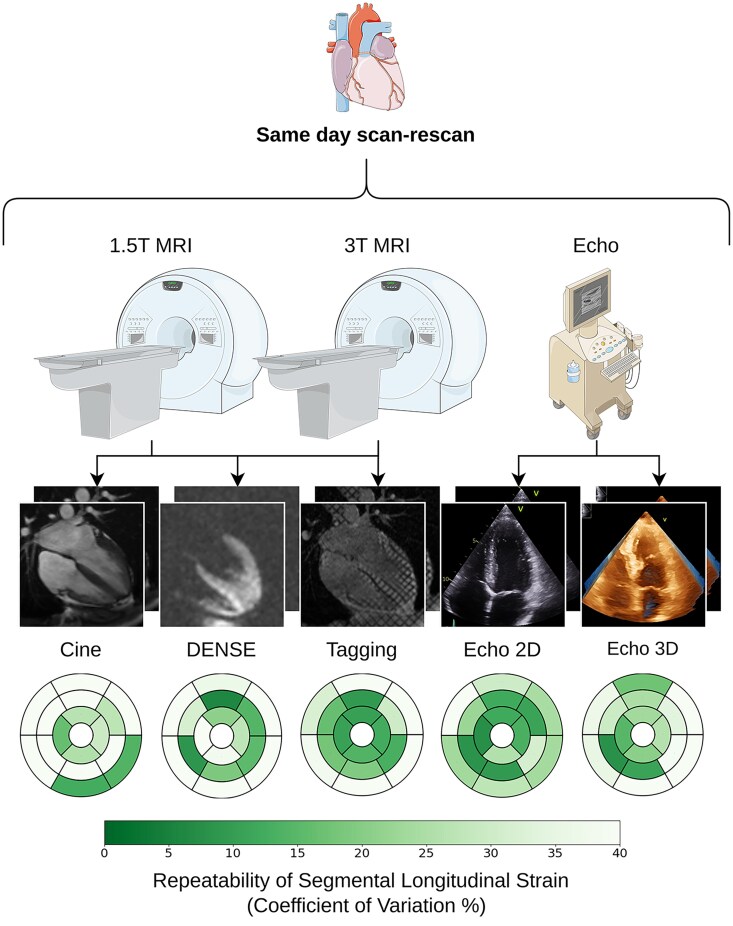
Twenty healthy subjects underwent same-day scan–rescan with eight imaging protocols: 1.5T and 3T CMR, with cine, DENSE and tagging sequences, as well as 2D and 3D echocardiography. Inter-scan repeatability of global and segmental strain measures was computed for each protocol. Shown are AHA left ventricular polar plots, colour-scaled according to the inter-scan repeatability of longitudinal strain for the given segment in terms of coefficient of variation. DENSE = Displacement Encoding with Stimulated Echoes. Icons from Servier Medical Art, provided by Servier, licensed under Creative Commons Attribution 4.0.

## Introduction

The assessment of myocardial function plays a key role in the diagnosis and prognosis of various cardiac disorders. Ejection fraction (EF), the relative change in ventricular volume during systole, is an important marker of function. However, abnormal left ventricular (LV) EF is a late manifestation of heart disease and may be preserved in cases such as heart failure with preserved ejection fraction.^1^

In contrast, myocardial strain represents the relative deformation of the heart muscle and is measured with respect to the axes of ventricular motion: Longitudinal, circumferential and radial. Longitudinal strain is of particular interest because it may indicate dysfunction of the vulnerable subendocardium before reduction in EF.^2^ Early detection of functional abnormalities is valuable in serial imaging applications such as cardio-oncology.^3^ Furthermore, strain is a powerful diagnostic marker, and predictor of outcomes in patients with a variety of cardiac disorders.^4,5^ Finally, segmental strain can provide a quantitative assessment of regional wall motion abnormalities,^2^ which may not be apparent with global parameters.

Despite its utility, strain has not been widely adopted into clinical use for several reasons. Firstly, the inter-scan repeatability of strain measurements is disputed.^6–11^ This metric is vital to quantify because clinical decisions are influenced by longitudinal changes in patient biomarkers. A higher repeatability allows smaller changes in strain to be detected and quantified. Although large changes in strain can be seen in patients with myocardial infarction, subtle changes of 1% are associated with increased risk of adverse events in cardio-oncology and heart failure.^12,13^ Furthermore, there are significant differences in measured strain depending on imaging modality, acquisition type and post-processing method,^14–21^ making it difficult to find reference values.

There are numerous strain imaging methods available, including echocardiography in two or three spatial dimensions, cine Cardiac Magnetic Resonance (CMR) and specialist CMR sequences, such as tagging, displacement encoding with stimulated echoes (DENSE), and strain-encoded imaging (SENC). Previous studies have not provided a comprehensive understanding of strain repeatability between methods. To address this, we performed a same-day scan–rescan of 20 healthy volunteers with eight imaging protocols. For each protocol, we assessed the inter-scan repeatability of both global and segmental strain.

## Methods

### Study design (consent)

This prospective study was approved by the institutional review board at Guy’s and St Thomas Hospital National Health Service Foundation Trust and the UK Health Research Authority. Exclusion criteria for the participants were CV symptoms or risk factors, regular medication, and personal or family history of CV disease. In total, 20 volunteers aged 34±8 were consecutively recruited and provided informed written consent; sample size was determined by scanner availability. The cohort consisted of 14 males (aged 33±6) and 6 females (aged 37±10) volunteers.

Participants underwent a same-day scan–rescan with eight imaging protocols: 1.5T and 3T CMR with cine, tagging, and DENSE sequences, as well as echocardiography in 2D and 3D. For CMR acquisitions, sequences were done in standard order: Localizers, Cine, Tagging, and DENSE. A 20-min break was taken between the first and second scans.

### Acquisition

Representative acquisition parameters for CMR protocols can be seen in Supplementary data online, table  *S1*.

### 2D & 3D Echocardiography

Scans were acquired with a Vivid E95 scanner (GE Healthcare). 2D Apical two-, three-, and four-chamber views were acquired for both scans, while parasternal short and long axes were acquired for the first scan only. The second scan had no short-axis view. Finally, a full 3D volume was acquired for both scans. Frame rate was 30 fps, and transducer frequency was 1.4 MHz.

### Cine CMR

Scans were acquired with an Aera 1.5T and a Biograph 3T scanner (Siemens), using a steady-state free precession sequence. Each scan included two-, three-, and four-chamber long-axis views. For the short axis, the Biograph scanner acquired basal, mid, and apical slices, while the Aera acquired 15 slices spanning the heart. Only three corresponding slices were used from the Aera for strain analysis to allow a fair comparison.

### CMR tagging

Scans were acquired with both scanners using a spatial modulation of magnetization sequence. Each scan included two-, three-, and four-chamber views and basal, mid, and apical slices in the short axis.

### CMR DENSE

Scans were acquired with both scanners. Each scan included two-, three- and four-chamber views and basal, mid, and apical slices in the short axis. Each view included a magnitude, x-encoded, and y-encoded phase image.

### Post-processing

Scans were analysed offline using a suitable software package for the imaging method. Due to the domain expertise required, a different observer was identified for each image type (Echo, Cine, Tag, and DENSE). Each observer analysed both scans of all 20 subjects for their respective image types. To avoid recall, scan and rescan analyses were performed in separate sessions. Image quality was assessed by the observers independently.

In 2D acquisitions, longitudinal strain was measured from long-axis views, while radial and circumferential strains were measured from short-axis views. Global strain values (GLS, GCS, and GRS) were defined as the frame-wise peak of the mean strain curve across the LV myocardium. Segmental strains were defined as the frame-wise peak of the mean strain within each LV segment, according to the 17-segment AHA model.^22^ All strain values are reported as percentage linear Lagrangian strain e:


$$e=100\text{\%}\times \frac{L_{ES}-L_{ED}}{L_{ED}}$$

where $L_{ED}$ is the length at end-diastole and $L_{ES}$ is the length at end-systole.

### 2D Echocardiography

Scans were analysed using Autostrain LV (TomTec) using a semi-automatic method. Endocardial contours were drawn automatically, checked by the observer, and adjusted if necessary. Contours define a region for speckle tracking between frames to compute myocardial strain.

### 3D Echocardiography

Scans were analysed using 4D LV analysis (TomTec) using a semi-automatic method. Endocardial contours were drawn automatically at the end-systolic frames of four projected views from the 3D acquisition. Contours and the derived 3D model are checked by the observer and adjusted if necessary. Contours are extruded to define a mid-myocardial region, over which speckle tracking is performed to calculate 3D strain.

### Cine CMR

Scans were analysed using CVI42 (Circle) using a semi-automatic method. Epi- and endocardial contours were drawn automatically at each frame, verified by the observer, and adjusted if necessary. Contours were used to automatically build a ventricular model, from which strain was analytically derived.^23^

### CMR Tagging

Scans were analysed using CIM Tag2D (University of Auckland), using a manual method.^24^ A 2D LV model was manually applied to the myocardium at ED, and then non-rigidly deformed to end-systole using the warped tag lines as a guide. Strain was computed automatically based on the model deformation between end-diastole and end-systole.

### CMR DENSE

Scans were analysed using CIM DENSE (University of Auckland), using a semi-automatic method.^24^ A 2D LV model was manually applied to the myocardium at ED. Strain was computed automatically by phase unwrapping.

### Statistical analysis

Statistical analysis was performed in Stata. Continuous data is represented as Mean ± SD if normally distributed, or as Median (IQR) otherwise. Normality was assessed with the Shapiro–Wilk test.

Inter-scan repeatability was assessed using the within-subject coefficient of variation (CoV) and its 95% confidence interval (CI). CoV was calculated using the root mean squared method:


$$COV(X,Y)=100\text{\%}\times \sqrt{\frac{2}{N}\overset{N}{\underset{i=1}{\sum }}\;{\left(\frac{x_{i}-y_{i}}{x_{i}+y_{i}}\right)}^{2}}$$

where $X=\{x_{1},x_{2},\ldots ,x_{N}\}$ and $Y=\{y_{1},y_{2},\ldots ,y_{N}\}$ are sets of paired measurements for the same set of subjects i∈{1,2,…,N}.^25^

The intraclass correlation coefficient (ICC) and its 95% CI were calculated based on a single rater, absolute agreement, two-way mixed effects model,^26^ and the mean bias and limits of agreement were calculated using Bland–Altman analysis.^27^ Error was defined as the mean of absolute differences (MAE) between two scans. ICC can underestimate repeatability when there is a low inter-subject variability (i.e. in healthy volunteers); thus, interpretations were made based on CoV, which is independent of inter-subject variance.^25^ Based on studies of cancer-therapy related cardiac dysfunction and heart failure,^12,13^ absolute strain differences of as little as 1% are associated with meaningfully increased risk. This corresponds to a CoV of 5% for typical strain, so we defined CoV agreement as excellent if CoV ≤ 5%, good if 5%<CoV ≤10%, fair if 10%<CoV ≤20% and poor if CoV > 20%.

## Results

### Demographics

The study cohort consisted of 20 healthy subjects; demographics and LV measurements are reported in *Table 1*. In total, 1640 2D slices or 3D volumes were processed, 19 of which could not be analysed; the reasons for which are detailed in supplementary data online, table  *S2*. Since both scans were required to evaluate repeatability, if one scan could not be analysed, then both were excluded.

**Table 1:** Demographics

Characteristic	Value
N	20 (14M)
Age (years)	34 ± 8
BMI (kg/m^2^)	24 ± 3
**LV Measurements (1.5T MRI)**	
End-diastolic volume (ml)	156.8 ± 30.7
End-systolic volume (ml)	67.1 ± 16.1
Stroke volume (ml)	89.7 ± 18.0
Ejection fraction (%)	57.4 ± 4.7
Mass (g)	87.8 ± 18.2

### Inter-scan repeatability

**Figure 1:**
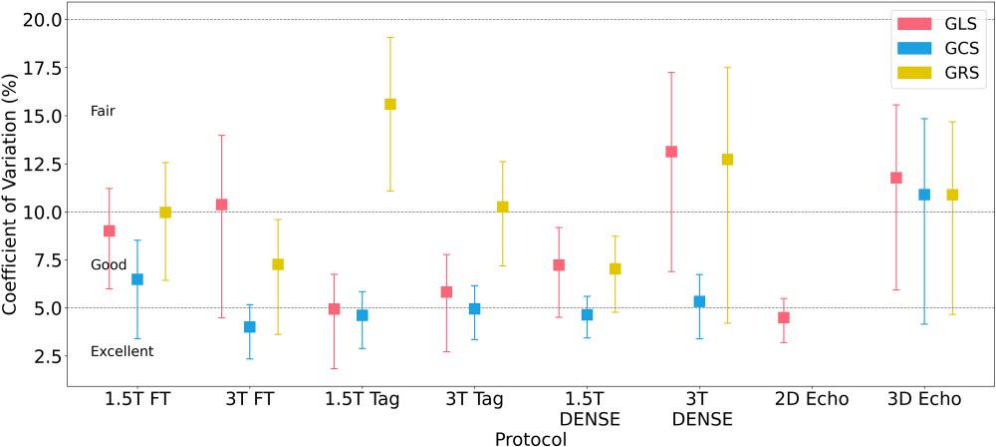
Inter-scan repeatability of imaging protocols. The mean coefficient of variation (CoV) values between two same-day scans are shown as dots for each protocol and strain measure. The 95% confidence intervals of the CoV are shown as error bars. GLS, Global longitudinal strain; GCS, Global circumferential strain; GRS, Global radial strain; DENSE, Displacement Encoding with Stimulated Echoes.

An overview of inter-scan repeatability across all eight protocols is shown in *Figure 1*, with detailed metrics reported in *Tables 2–4*. All methods achieved at least fair repeatability for global strain measures, with several reaching good or excellent performance depending on protocol and field strength. GCS and GRS repeatability could not be assessed in 2D Echo, due to the absence of parasternal views for the second scan.

**Figure 2:**
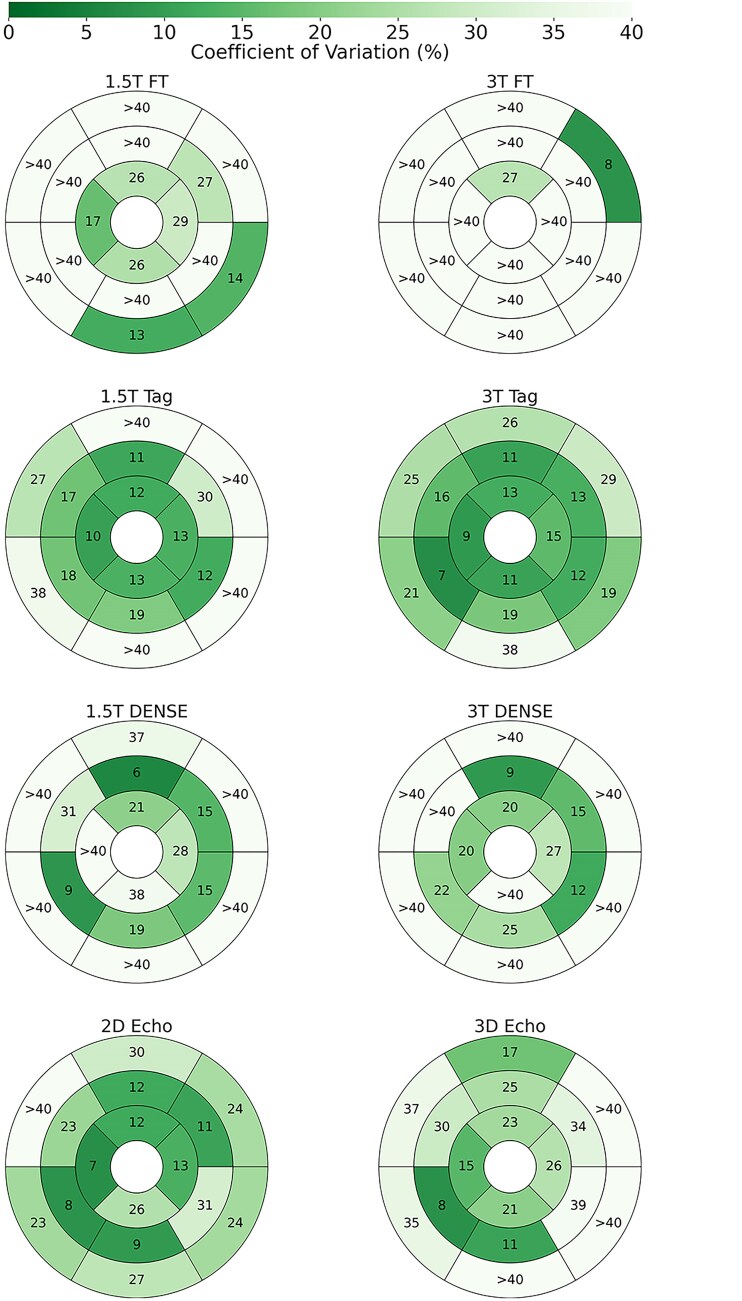
Repeatability of segmental longitudinal strain. Shown are AHA LV plots for each protocol, where the values and colour of the segments represent the inter-scan repeatability of longitudinal strain in terms of coefficient of variation (CoV).

**Figure 3:**
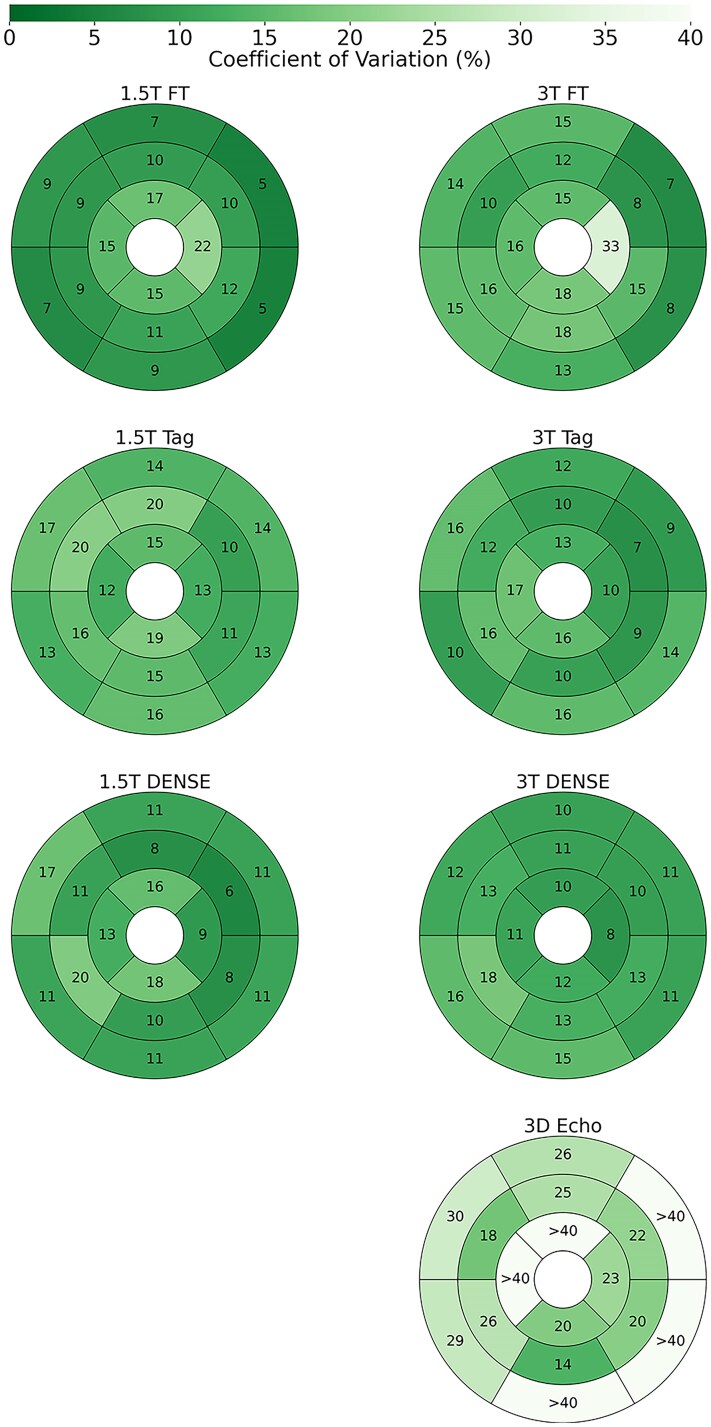
Repeatability of segmental circumferential strain. Shown are AHA LV plots for each protocol, where the values and colour of the segments represent the inter-scan repeatability of circumferential strain in terms of coefficient of variation (CoV).

**Figure 4:**
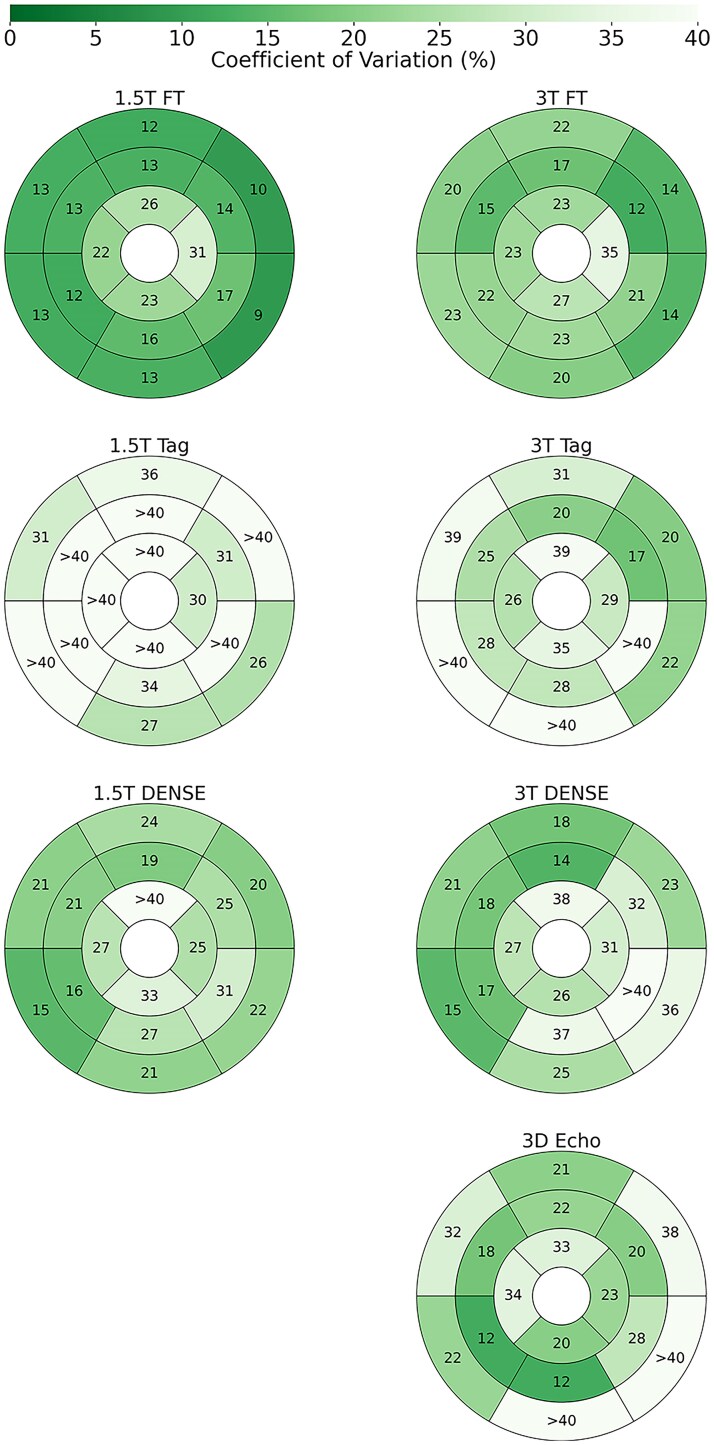
Repeatability of segmental radial strain. Shown are AHA LV plots for each protocol, where the values and colour of the segments represent the inter-scan repeatability of radial strain in terms of coefficient of variation (CoV).

**Table 2:** Inter-scan repeatability of GLS

Protocol	*n*	Bias (LoA)	Error (95% CI) ↓	ICC (95% CI) ↑	CoV (95% CI) ↓
1.5T FT	20	1.18 (−2.37, 4.73)	1.71 (1.12, 2.29)	0.62 (0.25, 0.83)	9.0 (6.0, 11.2)
3T FT	19	0.41 (−3.96, 4.77)	1.71 (1.04, 2.38)	0.50 (0.07, 0.77)	10.4 (4.5, 14.0)
1.5T Tag	17	0.04 (−2.33, 2.40)	0.91 (0.53, 1.29)	0.69 (0.34, 0.88)	4.9 (1.8, 6.7)
3T Tag	20	0.01 (−2.84, 2.86)	1.05 (0.61, 1.49)	0.72 (0.42, 0.88)	5.8 (2.7, 7.8)
1.5T DENSE	20	−0.13 (−2.73, 2.47)	1.12 (0.81, 1.44)	0.85 (0.66, 0.94)	7.2 (4.5, 9.2)
3T DENSE	20	0.04 (−4.58, 4.66)	1.66 (0.92, 2.39)	0.19 (−0.27, 0.57)	13.1 (6.9, 17.2)
2D Echo	20	0.09 (−2.38, 2.56)	1.09 (0.81, 1.37)	0.91 (0.78, 0.96)	4.5 (3.2, 5.5)
3D Echo	17	−0.41 (−4.89, 4.09)	1.63 (0.84, 2.42)	0.80 (0.53, 0.92)	11.8 (5.9, 15.6)

LoA, limits of agreement; CI, confidence interval; CoV, coefficient of variation; ICC, intraclass correlation coefficient; ↓ lower is better; and ↑ higher is better.

**Table 3:** Inter-scan repeatability of GCS

Protocol	*n*	Bias (LoA)	Error (95% CI) ↓	ICC (95% CI) ↑	CoV (95% CI) ↓
1.5T FT	20	0.35 (−2.68, 3.39)	1.17 (0.69, 1.64)	0.74 (0.46, 0.89)	6.5 (3.4, 8.5)
3T FT	19	−0.18 (−2.20, 1.84)	0.80 (0.50, 1.10)	0.89 (0.73, 0.95)	4.0 (2.3, 5.2)
1.5T Tag	20	0.42 (−2.29, 3.13)	1.08 (0.66, 1.50)	0.84 (0.64, 0.93)	4.6 (2.9, 5.8)
3T Tag	20	−0.35 (−3.07, 2.37)	1.19 (0.84, 1.54)	0.87 (0.69, 0.94)	5.0 (3.3, 6.2)
1.5T DENSE	20	0.52 (−1.91, 2.94)	1.17 (0.88, 1.46)	0.82 (0.60, 0.92)	4.6 (3.4, 5.6)
3T DENSE	20	0.08 (−2.80, 2.96)	1.20 (0.83, 1.57)	0.81 (0.59, 0.92)	5.3 (3.4, 6.7)
3D Echo	17	−0.41 (−5.24, 4.43)	1.84 (1.04, 2.65)	0.83 (0.59, 0.93)	10.9 (4.2, 14.8)

LoA, limits of agreement; CI, Confidence Interval; CoV, coefficient of variation; ICC, intraclass correlation coefficient; ↓ lower is better; and ↑ higher is better.

**Table 4:** Inter-scan repeatability of GRS

Scan	*n*	Bias (LoA)	Error (95% CI) ↓	ICC (95% CI) ↑	CoV (95% CI) ↓
1.5T FT	20	−1.18 (−9.44, 7.08)	3.35 (2.12, 4.59)	0.78 (0.52, 0.91)	10.0 (6.4, 12.6)
3T FT	19	0.34 (−6.26, 6.95)	2.35 (1.26, 3.45)	0.85 (0.65, 0.94)	7.3 (3.6, 9.6)
1.5T Tag	20	−2.48 (−14.65, 9.70)	5.55 (3.92, 7.19)	0.38 (−0.06, 0.70)	15.6 (11.1, 19.1)
3T Tag	20	0.61 (−8.97, 10.18)	4.06 (2.84, 5.28)	0.62 (0.26, 0.83)	10.3 (7.2, 12.6)
1.5T DENSE	20	−1.99 (−9.04, 5.07)	3.41 (2.41, 4.42)	0.83 (0.61, 0.93)	7.0 (4.8, 8.7)
3T DENSE	20	2.04 (−11.88, 15.95)	5.22 (2.94, 7.51)	0.62 (0.26, 0.83)	12.7 (4.2, 17.5)
3D Echo	17	1.55 (−11.42, 14.53)	4.49 (2.07, 6.92)	0.87 (0.67, 0.95)	10.9 (4.7, 14.7)

LoA, limits of agreement; CI, confidence interval; CoV, coefficient of variation; ICC, intraclass correlation coefficient; ↓ lower is better; and ↑ higher is better

#### Global longitudinal strain (GLS)

Repeatability for GLS was generally good but varied considerably between protocols. 2D Echo was strongest, with the lowest CoV (4.5%) and highest ICC (0.91), closely followed by 1.5T Tagging with CoV of 4.9% with the lowest error (0.91). 3T DENSE had the weakest repeatability, with the highest CoV (13.1%) and a markedly low ICC (0.19). Feature-tracking protocols had fair-to-good repeatability, with similar errors across 1.5T and 3T, though ICC was moderate (0.50–0.62). 3D Echo achieved fair reliability CoV (11.8%), but with a high ICC (0.80).

#### Global circumferential strain (GCS)

Repeatability for GCS was generally higher than for GLS, with all but one protocol achieving good or excellent reliability. 3T Feature tracking was the most repeatable, with the lowest error (0.80), highest ICC (0.89), and lowest CoV (4.0%), while FT at 1.5T was also good. Both tagging protocols had excellent repeatability, with ICCs ≥0.84 and CoV ≤5.0%. DENSE also performed well, with ICCs ≥0.81 and CoV ≤5.3% at both field strengths. 3D Echo had similar (fair) repeatability to the other measures, with the highest error (1.84) and CoV (10.9%).

#### Global radial strain (GRS)

In terms of GRS repeatability, 3D Echo and 1.5T DENSE were strongest, with the highest ICC (0.87), and lowest CoV (7.0%), respectively. In contrast, 1.5T tagging performed poorly, with the highest error (5.55), the lowest ICC (0.38), and the widest limits of agreement. Feature-tracking protocols, and 1.5T DENSE had good repeatability, with CoVs of 7–10% and ICCs of 0.78–0.85, while 3T DENSE was fair (12.7% CoV).

#### Segmental strains

Segmental strain repeatability (*Figures 2–4*) was highly variable for longitudinal and radial strains, with many segments and protocols having poor repeatability. For longitudinal strains, repeatability in the base was generally worse, while mid-segments for DENSE and Tagging were generally fair. Feature tracking had poor longitudinal strain repeatability in almost all segments. In terms of radial strains, tagging performed notably poorly, while feature tracking was generally fair. DENSE and 3D Echo varied from fair to poor. However, segmental circumferential strains were far more consistent, with most segments having fair repeatability for the MRI protocols, and poor repeatability for 3D Echo.

## Discussion

In serial imaging applications, small changes in strain value are associated with a meaningfully increased risk of cardiac disorders.^12,13,28^ Measures with low repeatability may distinguish groups with sufficiently large differences, but cannot reliably quantify the magnitude of this difference. It is therefore vital to understand the repeatability of strain imaging to interpret which changes are clinically relevant. To our knowledge, this is the first study to comprehensively evaluate the repeatability of strain using both CMR and echocardiography.

Inter-scan repeatability of global strain measures was excellent to fair across all protocols, suggesting that global strain measures are suitable for use in serial imaging settings. Feature tracking and DENSE at 1.5T were good across all global measures, while the repeatability of tagging declined for GRS. This is expected, given that previous work has found poor inter-study reproducibility of tagging when measuring GRS.^29^ Limitations in tagging image resolution mean that often only a single grid square can fit within the myocardium, making assessments of radial motion unreliable. 2D echocardiography offered the best GLS repeatability of any protocol, while 3D Echo demonstrated lower, but still fair repeatability and could benefit from further improvements in robustness.

Previous literature has examined several of these protocols in relative isolation, on both healthy subjects and consecutive patients. Although study designs vary, our results are generally consistent with these prior findings. For example, one study reported 2D Echo inter-scan GLS CoV of 5.4% (same-day test–retest) on consecutive patients,^30^ and another reported 5.0% (∼6-day scan–rescan) in healthy subjects,^11^ both values close to our 4.5%. In the latter study, feature-tracking GLS CoV was 5.6%, somewhat lower than our findings, likely due to different FT software. A separate ∼14-day scan–rescan study reported FT-derived GLS CoV of 10.4%,^9^ which is very similar to our findings despite differing study design. Finally, a ∼40-day scan–rescan that included healthy subjects and heart failure patients reported FT GLS CoV of 10.8% (agreeing with our results) and Tagging GLS CoV of 9.4%, which is somewhat worse than in our study; that study also reported poor repeatability for mean segmental longitudinal strain.^8^ Our study provides a broader picture of strain repeatability by directly comparing multiple protocols and reporting in-depth metrics for each, and these results align with the limited previous literature. Furthermore, repeatability results are similar across healthy and patient populations, suggesting that our results may translate to a patient setting.

Segmental strain may offer a more granular assessment of myocardial function, with the potential to identify localized abnormalities that may not be apparent with global measures. However, by measuring a small region of the LV, achieving repeatability is challenging for segmental strain. Feature tracking had poor repeatability when measuring segmental longitudinal strain. This is most likely due to through-plane motion, which greatly affects regional assessments of deformation, but can be mitigated by averaging to a global measure. Tagging and DENSE often had fair repeatability for mid and apical measures but were usually poor in the base. The base of the ventricle has the greatest longitudinal velocity during systole, making motion assessments especially difficult in protocols with limited temporal resolution. Both 2D and 3D echocardiography also had worse repeatability in the base, but 2D Echo had good or fair segmental longitudinal strain repeatability in most apical and mid-segments. This may be due to more signal attenuation at the base in Echo acquisitions. Segmental radial strain had poor to fair repeatability with feature tracking, while in general tagging and DENSE were poor. FT benefits from the relatively small radial distance between the endo- and epicardium. This proximity reduces the likelihood that tracked features will experience a relative shift due to through-plane motion, while tagging and DENSE suffer due to limited spatial resolution. 3D Echo had generally fair repeatability when measuring segmental radial strain.

Circumferential strain had markedly better repeatability than longitudinal and radial strain for all protocols except 3D echocardiography. This is true when measured globally and for almost all segments when measured regionally. While GLS is a more sensitive and earlier marker of myocardial dysfunction,^31^ circumferential strain was better suited to segmental analysis. In a clinical setting, this could reveal regional abnormalities in circumferential motion, potentially offering improved diagnostic precision.

These findings should be interpreted within the context of this study's limitations. The cohort consisted of only healthy subjects; inter-scan repeatability of strain may be different in patients. This is because analysis tools may not be as robust in the presence of cardiac dysfunction, and co-morbidities and medications could affect same-day strain measurements considerably. Furthermore, factors such as the scanning protocol, analysis software, and observer were all controlled in our study, but may vary between scans in clinical practice. We did not examine 3D isotropic MRI acquisitions, since these are not yet feasible in clinical practice. Isotropic resolution may improve repeatability across the different axes in the future. A power calculation for the number of subjects was not performed, due to a lack of previous data on strain scan–rescan reproducibility across different modalities. The number of subjects was determined by practical limitations such as scanner availability and tolerance to many scans, but our data can be used to perform power calculations in subsequent studies. Finally, by comparing same-day scans, we measured repeatability of the imaging methods themselves, but we controlled for potential biological changes that occur between longitudinal scans.

In conclusion, this study found that both CMR and Echocardiography had excellent to fair repeatability for global strain measures but were often poor for segmental measures. Using feature tracking at 1.5T, relative GLS changes exceeding 11.2% are unlikely to be caused by measurement variability alone; this figure is 5.5% for 2D echocardiography. Future work should target reproducibility in cardiomyopathies and improve repeatability of segmental strain measures, in particular, longitudinal strain. This would enable a robust detection of subclinical cardiac dysfunction at a granular level.

## Supplementary Material

qyaf144_Supplementary_Data

## Data Availability

The data underlying this article will be shared on reasonable request to the corresponding author.

## Lead author biography

Andrew Bell is a PhD student at King’s College London, previously earning his MSc in computer science at the University of Warwick. He is interested in advancing image analysis for the quantification of heart muscle function.
