# Supplementary material for: The strain-8 study: a multimodal scan–rescan assessment of myocardial strain repeatability
Source: Eur Heart J Imaging Methods Pract. 2025 Nov 14;3(4):qyaf144. doi: 10.1093/ehjimp/qyaf144 (PMC12671395; doi:10.1093/ehjimp/qyaf144)
Supplement: qyaf144_Supplementary_Data [file qyaf144_supplementary_data.docx]

# Supplemental Table 1: CMR Acquisition Parameters

| **Scanner** | **Image Type** | **FOV (mm)** | **Flip Angle (degrees)** | **Image Dims (px)** | **Pixel Spacing (mm)** | **Slice Thickness (mm)** | **TE (ms)** | **TR (ms)** |
| --- | --- | --- | --- | --- | --- | --- | --- | --- |
| 1.5 | Cine | 255x340 | 53 | 144 x 192 | 1.77 x 1.77 | 8 | 1.11 | 34.45 |
|  | DENSE | 200x200 | 15 | 80x80 | 2.50 x 2.50 | 8 | 1.08 | 15 |
|  | Tag | 276x340 | 14 | 208 x 256 | 1.33 x 1.33 | 6 | 3.92 | 49.56 |
| 3T | Cine | 304x360 | 50 | 216 x 256 | 1.41 x 1.41 | 8 | 1.49 | 44.07 |
|  | DENSE | 200x200 | 15 | 80x80 | 2.50 x 2.50 | 8 | 1.08 | 15 |
|  | Tag | 320x257 | 10 | 224 x 180 | 1.43 x 1.43 | 6 | 2.57 | 59.51 |

Supplemental Table 2: Excluded Data

| Protocol | Subject | Scan | View | Reason |
| --- | --- | --- | --- | --- |
| 1.5T Tagging | 6 | A | 3 Chamber | Planning |
| 1.5T Tagging | 9 | A | 2 Chamber | Artefact |
| 1.5T Tagging | 14 | A | 4 Chamber | Planning |
| 1.5T Tagging | 14 | B | 3 Chamber | Artefact |
| 3T Cine | 19 | A,B | All | Missing |
| 3D Echo | 2 | B | All | Quality |
| 3D Echo | 3 | A,B | All | Quality |
| 3D Echo | 5 | A,B | All | Quality |
